# Mapping within‑field variability of soybean evapotranspiration and crop coefficient using the Earth Engine Evaporation Flux (EEFlux) application

**DOI:** 10.1371/journal.pone.0235620

**Published:** 2020-07-09

**Authors:** Luan Peroni Venancio, Fernando Coelho Eugenio, Roberto Filgueiras, Fernando França da Cunha, Robson Argolo dos Santos, Wilian Rodrigues Ribeiro, Everardo Chartuni Mantovani

**Affiliations:** 1 Department of Agricultural Engineering, Federal University of Viçosa, Viçosa, Minas Gerais, Brazil; 2 Department of Forest Engineering, Federal University of Santa Maria, Cachoeira do Sul, Rio Grande do Sul, Brazil; 3 Department of Rural Engineering, Federal University of Espírito Santo, Alegre, Espírito Santo, Brazil; Hellenic Agricultural Organization - Demeter, GREECE

## Abstract

Accurate information about the spatiotemporal variability of actual crop evapotranspiration (ETa), crop coefficient (K_c_) and water productivity (WP) is crucial for water efficient management in the agriculture. The Earth Engine Evapotranspiration Flux (EEFlux) application has become a popular approach for providing spatiotemporal information on ETa and Kc worldwide. The aim of this study was to quantify the variability of water consumption (ETa) and the K_c_ for an irrigated commercial planting of soybeans based on the EEFlux application in the western region of the state of Bahia, Brazil. The water productivity (WP) for the fields was also obtained. Six cloud-free images from Landsat 7 and 8 satellites, acquired during the 2016/17 soybean growing season were used and processed on the EEFlux platform. The ETa from EEFlux was compared to that of the modified FAO (MFAO) approach using the following statistical metrics: Willmot’s index of agreement (d-*index*), root mean square error (RMSE), mean absolute error (MAE) and mean bias error (MBE). The K_c_ from EEFlux was compared to the K_c_ used in the soybean field (K_c_ FAO-based) and to the K_c_ values obtained in different scientific studies using the d-*index*. A similar procedure was performed for WP. Our results reveal that EEFlux is able to provide accurate information about the variability of ETa and the K_c_ of soybean fields. The comparison between ETa EEFlux and ETa MFAO showed good agreement based on the d-*index*, with values of 0.85, 0.83 and 0.89 for central pivots 1, 2 and 3, respectively. However, EEFlux tends to slightly underestimate ETa. The K_c_ EEFlux showed good accordance with the K_c_ values considered in this study, except in phase II, where a larger difference was observed; the average WP of the three fields (1.14 kg m^-3^) was higher than that in the majority of the previous studies, which is a strong indicator of the efficient use of water in the studied soybean fields. The study showed that EEFlux, an innovative and free tool for access spatiotemporal variability of ETa and Kc at global scale is very efficient to estimate the ETa and Kc on different growth stages of soybean crop.

## Introduction

One of the great current and future challenges worldwide is the production of more food to serve a growing population, which in 2050 can reach 9.73 billion people [1], in a scenario with less water available for agriculture. Thus, the appropriate management of water resources is extremely important [2]. In the specific case of irrigated agriculture, management is even more important because it represents the largest user of freshwater in the world, being responsible for approximately 70% of freshwater consumption, which is withdrawn from surface water and groundwater resources [1]. Among some of the reasons for the this high consumption, there is the low efficiency of the majority of the world existing irrigation systems, which is, on average, of only 56% [3], although there are systems with a higher irrigation efficiency, such as drip and central pivot. In addition, many irrigators do not adopt a method or tool monitor the crops water consumption. Zhang et al. [4] mentioned that, currently, irrigation depth is still often applied based on experience instead of science. To overcome this issue and consequently improve water use in agriculture, Kamali & Nazari [5] reported that two main strategies can be used: (i) upgrading operationally inefficient irrigation systems and (ii) improving irrigation planning and management.

With the current technological level of the irrigation industries, a lot of efficient equipment are available, so from an operational point of view, the first strategy can be relatively easy to reach. However, there a cost of acquisition and implantation of the system which need be carefully analyzed. The second point, however, is slightly more complex, as it is necessary to accurately quantify water consumption by crops, which, in turn, depends on the dynamic relationships between the soil-plant-atmosphere (SPA) system. The traditional ways to quantify the water consumption by crops are, mainly, the FAO (Food and Agriculture Organization) approaches (K_c_ single and dual) [6], weighing lysimeters, eddy-covariance (EC) systems and Bowen ratio [7]. These methodologies, although presenting many advantages, are locally based and, due to variations in climatic characteristics, have limitations when used on large areas [5]. In other words, these methodologies do not provide information on water consumption variability in the cultivation area. Thus, the location and quantity of the equipment (e.g., weather station, lysimeters and EC) must be strategic to have a reasonable representation of the whole area.

On the other hand, satellite-based surface energy balance models are a viable alternative to assess crop water consumption as well as to obtain K_c_. Remote sensing (RS) has a strong advantage in spatial data acquisition since the information is acquired spatially [8] for large areas in a systematic way, with lower time and cost [5]. Mapping EvapoTranspiration at high Resolution using Internalized Calibration (METRIC) [9,10], which is capable of accurately estimating the water consumption by crops, has a very consistent physical basis and is one of the best models developed over the last few years; therefore, it has been successfully applied in many countries [8,11–13].

One of the problems related to the applicability of the energy balance models, targeted for final users, is the need for background knowledge in the physics of radiation [14,15]. METRIC users, for example, need to accumulate and assemble a variety of layers, including satellite images, land cover maps, terrain, local climate and soil maps from different sources and platforms, and there might be a significant amount of preprocessing required for the different layers before applying the algorithm [16]. Data entry and manipulation can be the most time-consuming phase of this process [17]. However, the Earth Engine Evapotranspiration Flux (EEFlux) platform has recently developed a METRIC version that operates on the Google Earth Engine (GEE) system [16,18]. Thus, the data entry and manipulation were automated, and the ET estimation process became faster than what was achieved with previous methods.

The goal of EEFlux development is the provision of on-demand estimates of the spatial distribution of water consumption by vegetation with 30 m spatial resolution, which are applicable to the 1984-present recording period for thermal-equipped Landsat imagery [19]. EEFlux also provides the rapid generation of intermediate products, such as surface temperature (Ts), normalized difference vegetation index (NDVI) [20] and albedo for a given Landsat scene, which may be useful for other applications besides ET [16]. EEFlux products can be considered "ready-to-use remote sensing products". Thus, this ease of use has motivated the scientific community to use these products instead of performing laborious imaging processing to generate similar products [15,21].

Some works have been conducted recently using EEFlux products [17,22–25]. Costa et al. [17] for example, found results satisfactory for estimate the spatial variability quantification of maize water consumption in Brazil [17]. Ayyad et al. [22], evaluated ETa from EEFlux in Egyptian agricultural areas of the Nile Delta and the Nile Valley and verified that their estimations produce overestimations for the ETa values. Khan et al. [23] compared ETa from EEFlux with eddy covariance measurements at four sites with five annual crops, and verified reasonable agreement between data. On the other hand, none of them has been carried out to soybean irrigated fields, and analyzed the variability of the crop evapotranspiration and crop coefficient, being our study the first. Study like the one being proposed is necessary to help in a better water use in agriculture, being also its important the knowledge about the water productivity (WP), a quantitative term used to define the relationship between agricultural output and the amount of freshwater involved in crop production (in kg m^-3^ or kg ha^-1^ mm^-1^), which is a measure of the efficiency of water use [26,27].

Based on the importance of improving soybean irrigation planning and management, the present study aims to quantify the variability in water consumption (ETa) and the K_c_ for an irrigated commercial site of soybean planting based on the METRIC algorithm of the Google EEFlux application in the western region of the state of Bahia, Brazil. The specific objective includes a comparison of the spatial estimates of ETa EEFlux with the modified FAO method estimates, which is a verified and established method for crop evapotranspiration estimates in Brazil employed in the study area. In addition, we also aim to assess the water-use efficiency (WUE) in the soybean fields with the estimation of WP.

## Materials and methods

### Site description

**Fig 1:**
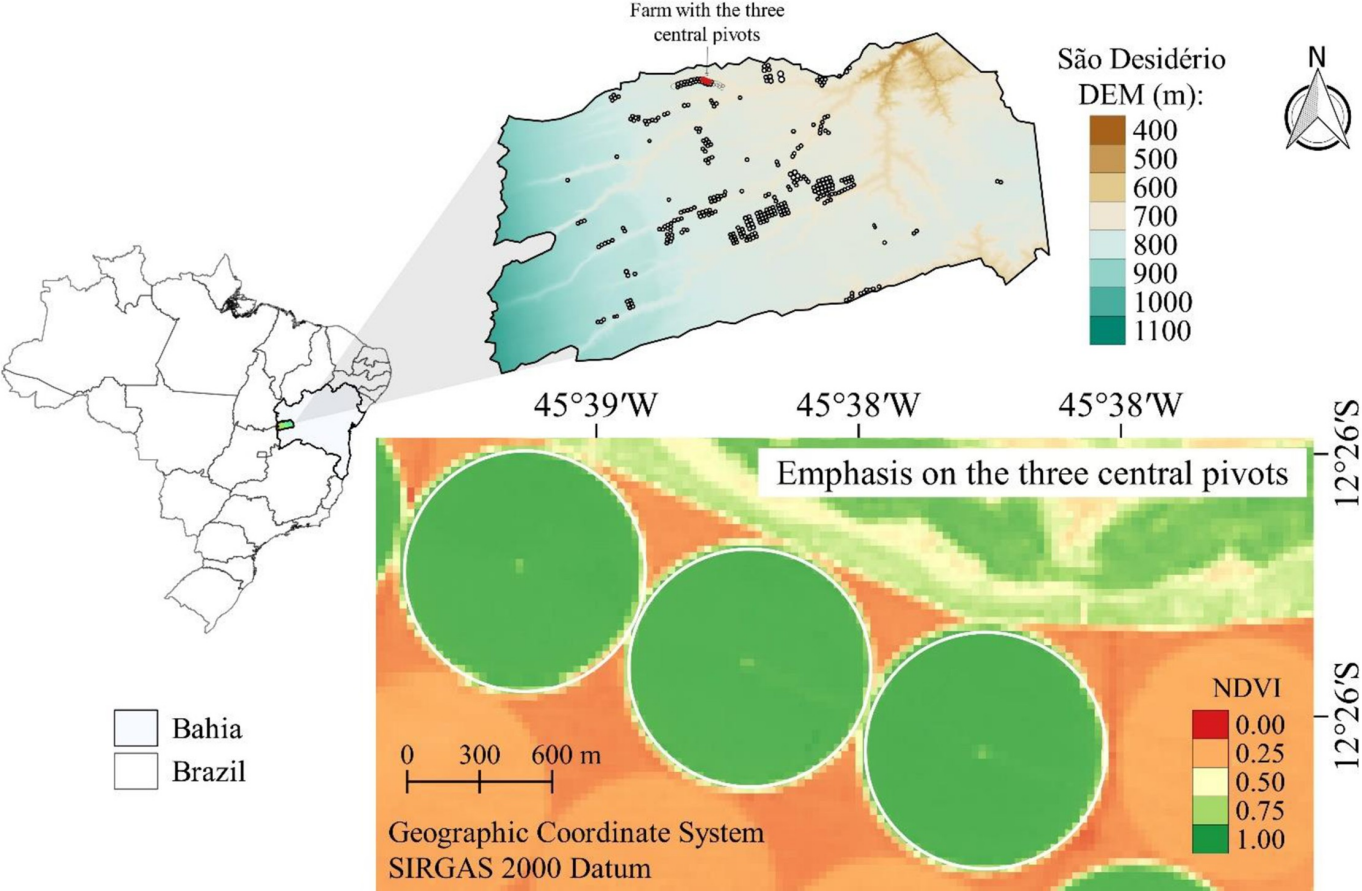
Location of study area and identification of the central pivots. DEM is the digital elevation model from the Shuttle Radar Topography Mission (SRTM) with a 30m spatial resolution downloaded from http://www.webmapit.com.br/inpe/topodata/. NDVI is the Normalized Difference Vegetation Index.

The study was carried out on a traditional farm cropped with soybean (*Glycine max* L.) located in the municipality of São Desidério, in the western region of the state of Bahia, Brazil. Soybean is the main commercial oilseed crop and one of the main sources of vegetable oil and vegetable protein in the world [28,29] and is also the most important crop of Brazil [30]. This region stands out in the Brazilian and global map of agribusiness, being responsible to produces 100% of the soybean crop of the state, and 65% of its cultivated areas are occupied by this oilseed. In the 2017/18 harvest, the occupied area corresponded to 1.6 million hectares, with a production of 6.3 million tons and yield of 3.96 Mg ha^-1^ [31].Three central pivots with an area of 80 ha each were used. The central pivots were located in a rectangle, bound by the coordinate pairs 12°26'45''S-45°39'28''W and 12°25'40''S-45°37'48''W, and they had an average altitude of 750 m above sea level (Fig 1). According to Köppen’s climatic classification [32], the climate of the region is tropical (Aw), with a rainy season in summer and dry winter, with an annual normal precipitation in the region of 1003.4 mm [33], concentrated in the rainy season (October to April). The region is characterized by having one of the highest concentrations of central pivots in Brazil [34] and being a great producer of cotton, soybean, and maize [35].

### Field data

Field data refer to the soybean (*Glycine max* L.) crop, meteorological conditions, and ETa collected during the 2016/2017 soybean growing season. The soybean data used in this study refer to the cultivar name, row and seed spacing, sowing and harvest date, cycle duration and yield mentioned in Table 1. Harvests were performed using harvesting machines, and the three central pivots were harvested together, which gave an average yield shown in Table 1.

**Table 1:** Data referring to the soybean (*Glycine max* L.) cropped in the central pivots 1, 2 and 3 in the crop season 2016/2017.

Cultivar -	Spacing (seeds × row)	Sowing date -	Harvest date -	Cycle (days)	Yield (kg ha^-1^)
Monsoy—M8349 IPRO	0.2 × 0.45 m	24/10/2016	04/03/2017	131	4,042

Meteorological data acquired included minimum air temperature (Tmin, °C), maximum air temperature (Tmax, °C), wind speed at 2 m height (WS, m s^-1^), radiation (Ra, MJ m^2^ d^-1^), relative humidity (RH, %) and rainfall (mm). These data were obtained from an automatic meteorological station located near the central pivots. The soybean ETa was also measured, calculated using the modified FAO method (MFAO) [36,37]. The temporal variations in these meteorological data over the season are presented in the results and discussion.

### Actual crop evapotranspiration (ETa) estimates

#### Modified FAO method

Soybean ETa was estimated by the modified FAO method (MFAO), an approach verified in Brazil in large irrigated commercial areas and abroad, through the irrigation platform “IRRIGER Connect” from Valmont Industries [38]. This methodology is also largely applied in scientific research [39–41]. The MFAO method is derived from the single-crop coefficient empirical method (Allen et al., 1998; Doorenbos and Pruitt, 1977), which is one of the most commonly used methods for irrigation water management [42,43] (Eq 1).

$$\mathrm{ETa}=\mathrm{ETo}\times K_{c}\times K_{S}\times K_{L}$$ (1)

where ETa is the actual crop evapotranspiration (mm d^-1^), ETo is the grass reference evapotranspiration according to the FAO Penman-Monteith method approach [6] (mm d^-1^), K_c_ is the crop coefficient, K_S_ is the water stress coefficient [37,40] and K_L_ is the localized water application coefficient [44]. The value of K_L_ is 1 when the whole cultivated area is wetted by an irrigation system (e.g., areas are irrigated by central pivots).

The K_c_ values used in the MFAO method for soybean ETa determination are derived from the values recommended by the FAO-56 approach [6]. Table 2 shows the following K_c_ values based on the grass-reference evapotranspiration used.

**Table 2:** Information about soybean K_c_ used in the MFAO method for ETa estimates.

FAO phenological phase	Phenological phase No.	K_c_ value	Length (days)	K_c_ calculation
Initial	I	0.35	15	Constant
Development	II	-	35	Linear interpolation
Mid-season	III	1.00	52	Constant
Late-season	IV	-	30	Linear interpolation
End	-	0.70	-	Constant

Kc during the crop development phase: linear interpolation between Kc values of the I and III phenological phases. Kc during the late-season phase: linear interpolation between Kc values of the III and Kc end.

The K_S_ is used to incorporate the water stress effect on reducing crop transpiration, and the daily K_S_ estimation in the root zone [37] is computed as follows (Eq 2):

$$K_{S}=\frac{\ln(1+\mathrm{CSWS})}{\ln(1+\mathrm{SWS})}$$ (2)

where SWS is the total soil water storage (mm) and CSWS is the current soil water storage (mm).

#### Earth Engine Evapotranspiration Flux (EEFlux)

The Earth Engine Evapotranspiration Flux (EEFlux) is patterned after the operational stand-alone METRIC (Mapping Evapotranspiration at High Resolution with Internal Calibration) model [9,10]. EEFlux is a full surface energy balance model that produces estimates of net radiation (Rn), sensible heat flux (H) and soil heat flux (G) [16,18]. The ETa is estimated as a residual of the surface energy balance [9,10], according to Eq 3.

LE=Rn−H−G (3)

where LE is the latent heat flux—spent energy in the evapotranspiration process (W m^-2^), Rn is the net radiation (W m^-2^), G is the soil heat flux, (W m^-2^) and H is the sensible heat flux (W m^-2^).

The LE is estimated at the exact satellite overpass time for each pixel. The ETa is then calculated by dividing LE by the latent heat of vaporization, according to Eq 4.

$${\mathrm{ET}}_{\mathrm{inst}}=\frac{\mathrm{LE}}{{\lambda \rho }_{w}}\times 3600$$ (4)

where ET_inst_ is the instant evapotranspiration (mm h^-1^), 3600 converts seconds to hours, λ is the latent heat of vaporization (J kg^-1^) and ρ_w_ is the density of water (∼ kg 1000 m^-3^).

Later, the fraction of the reference evapotranspiration (ETrF) was calculated for each pixel by the ratio of the computed ETinst to the instantaneous alfalfa reference evapotranspiration (ETr), according to Eq 5, and it was used as a vehicle to extrapolate ET from the instantaneous passage of the satellite to a 24-h period. The ETrF is a crop coefficient relative to ETr (K_cr_) since alfalfa is the reference crop adopted in METRIC [45].

$${\mathrm{ET}}_{r}F=\frac{{\mathrm{ET}}_{\mathrm{ins}}}{{\mathrm{ET}}_{r}}$$ (5)

Thus, the daily ETa is estimated by multiplying ETrF values for each individual pixel by daily ETr, computed from local or gridded weather data, assuming consistency between the ETrF at the time of the satellite passage and the ETrF for the 24-hour period, as follows.

$${\mathrm{ET}}_{a}={\mathrm{ET}}_{\mathrm{inst}}\times {\mathrm{ET}}_{r}F$$ (6)

It is important to note that both EEFlux and METRIC applications utilize the alfalfa reference evapotranspiration (ETr) [46] instead of the ETo to estimate the daily ETa [16]. However, in the central pivots of this study, the ETo was used instead of the ETr to estimate the ETa. Thus, part of the difference between the estimates (EEFlux and MFAO) that will be verified can be attributed to this, although the difference between the derived ETa to ETr or ETo can be considered small, due to K_c_ value adjustments [6].

To quantify the variability of ETa and K_c_, six Landsat 7 and 8 satellite images were used and processed on Earth Engine Evapotranspiration Flux (EEFlux/METRIC version 0.20.2; https://eeflux-level1.appspot.com/). Table 3 shows the Landsat 7 and 8 image information, as well as information related to the soybean crop: days after sowing (DAS) and soybean growth stages for the image dates. In addition, Table 4 shows details of the agrometeorological conditions on the date of the images collections.

**Table 3:** Details for Landsat satellite used (7 or 8), date and Day of The Year (DOY) of acquisition of the image, Days After Sowing (DAS), soybean growth stages and FAO phenological stage of soybean (*Glycine max* L.), considering Monsoy—M8349 IPRO cultivar growth.

Landsat	Acquisition		DAS	Soybean growth stages	FAO phenological stage
	Date	DOY			
7	Oct. 26, 2016	300	002	VE—Emergence	I—Initial
8	Nov. 03, 2016	308	010	V1—First trifoliolate	I—Initial
8	Jan. 06, 2017	006	074	R4—Full pod	II—Development
8	Jan. 22, 2017	022	090	R5—Beginning seed	III—Mid-season
7	Feb. 15, 2017	046	114	R6—Full seed	IV—Late-season
8	Feb. 23, 2017	054	122	R7—Beginning maturity	IV—Late-season

**Table 4:** Agrometeorological conditions on the date of the images collections.

Acquisition date	Tm (°C)	RH (%)	WS (m s^-1^)	Ra (MJ m^-2^ d^-1^)	P (mm)	ETo (mm d^-1^)
Oct. 26, 2016	26.8	50.0	2.1	30.6	0.0	6.8
Nov. 03, 2016	27.3	39.0	3.4	31.8	0.0	8.4
Jan. 06, 2017	27.6	49.7	0.3	26.9	0.0	4.8
Jan. 22, 2017	25.5	70.4	2.2	29.4	0.0	5.9
Feb. 15, 2017	22.7	79.1	1.8	21.4	0.0	3.8
Feb. 23, 2017	24.7	71.3	1.7	29.2	0.0	5.4

Tm—mean air temperature. RH—relative humidity. WS—wind speed at 2 m height. Ra—extraterrestrial radiation. P—rainfall. ETo—grass reference evapotranspiration.

### Crop coefficient (K_c_)

The K_c_ derived from EEFlux, which is an alfalfa-based crop coefficient, was compared to the K_c_ adopted in soybean fields (FAO-based Kc) and with those derived from studies performed in the United States and Brazil. Those K_c_ values obtained based on grass were converted into alfalfa-based K_c_ through the means of their division by a conversion factor (K_ratio_) calculated using Eq 7, as recommended in the FAO 56 approach [6].

$$K_{\mathrm{ratio}}=1.2\left[0.04\;(\mathrm{WS}-2)-0.04\;({\mathrm{RH}}_{\min}-45)\;(\frac{h}{3})\right]$$ (7)

where WS is the wind speed at 2 m height (m s^-1^), RH_min_ is the relative humidity (%) and h is equal to 0.5, which is the standard height for the alfalfa reference.

In addition, the K_c_ values were strategically chosen to coincide with the image date or near to it, allowing their comparison with K_c_ EEFlux. The K_c_ values after conversion to the alfalfa reference are presented in Table 5.

**Table 5:** Soybean crop coefficient (K_c_) based on alfalfa reference evapotranspiration obtained in different phenological stages established by the FAO (I—initial; II—development; III—mid-season; and IV—late-season) along with the respective days after sowing (DAS), calculated in irrigated areas by central pivots in the United States and Brazil.

Reference—Region	K_c_ values (Approximate DAS)				
	I	II	III	IV-1	IV-2
Allen et al. [6]—USA^a^	0.29 (002)	0.83 (074)	0.83 (090)	0.72 (114)	0.61 (122)
Singh & Irmak [47]—USA^b^	0.24 (004)	0.86 (068)	1.11 (084)	0.75 (116)	0.58 (124)
Suyker & Verma [48]–USA	0.17 (010)	0.79 (075)	0.91 (090)	0.62 (110)	0.25 (120)
Monteiro & Sentelhas [49]—BRA^c^	0.25 (001)	0.62 (064)	0.95 (093)	0.66 (110)	0.66 (120)

^a^ Kc adopted in the soybean fields after their conversion to alfalfa-based Kc.

^b^ Kc derived from alfalfa-reference evapotranspiration (ETr), so conversion was not necessary.

^c^ the work does not specify whether the areas were irrigated.

### Water productivity (WP) and NDVI-K_c_ relationship

The WP, also denominated as water use efficiency (WUE), is a quantitative term used to define the relationship between agricultural output and the amount of freshwater involved in crop production (in kg m^-3^ or kg ha^-1^ mm^-1^) [26,27,50]. Crop WP in this study was expressed in terms of actual evapotranspiration (WP_ETa_) according to Eq 8 [27].

$$\mathrm{WP}=\frac{\mathrm{Ys}}{\sum {\mathrm{ET}}_{a}}$$ (8)

where WP is the water productivity (kg ha^-1^ mm^-1^ or kg m^-3^), Ys is the soybean yield (kg ha^-1^) and ΣETa is the sum of ETa over the season calculated by the MFAO method (mm or m^3^ ha^-1^).

The relationship between NDVI and the Kc, named NDVI-Kc relationship, was establish by means a simple linear regression. NDVI was chosen because, similar to ETa and K_c_, it is available "ready-to-use" in the EEFlux platform. Furthermore, the NDVI is probably the most frequently used vegetation index for crop biophysical parameter access. Eq 9 and the K_c_ are both derived from the EEFlux platform.

$$\mathrm{NDVI}=\frac{\left(\rho_{\mathrm{NIR}}-\rho_{\mathrm{red}}\right)}{\left(\rho_{\mathrm{NIR}}+\rho_{\mathrm{red}}\right)}$$ (9)

where ρ_NIR_ and ρ_red_ refer to the reflectance of the near-infrared and red spectral bands, respectively.

### Variables, maps, and statistical analysis

The temporal and spatial dynamics of ETa and K_c_ have been developed. In addition, the temporal and spatial distribution of NDVI was obtained to assess soybean development over the season. Then, the descriptive statistical (i.e., average, minimum and maximum values) sample was obtained by a boxplot for the NDVI, ETa and K_c_ based on EEFlux data for each of the seven Landsat images acquired over the season. Later, the ETa obtained through EEFlux was compared to the ETa observed in the field (MFAO) using the following statistical metrics: Willmot’s index of agreement (d-*index*) [51], root mean square error (RMSE), mean absolute error (MAE) and mean bias error (MBE). The K_c_ was compared using only the d*-index*. The d*-index* value of 1 indicates a perfect match, and 0 indicates no agreement at all.

## Results and discussion

### Climatic conditions and irrigation depth

**Fig 2:**
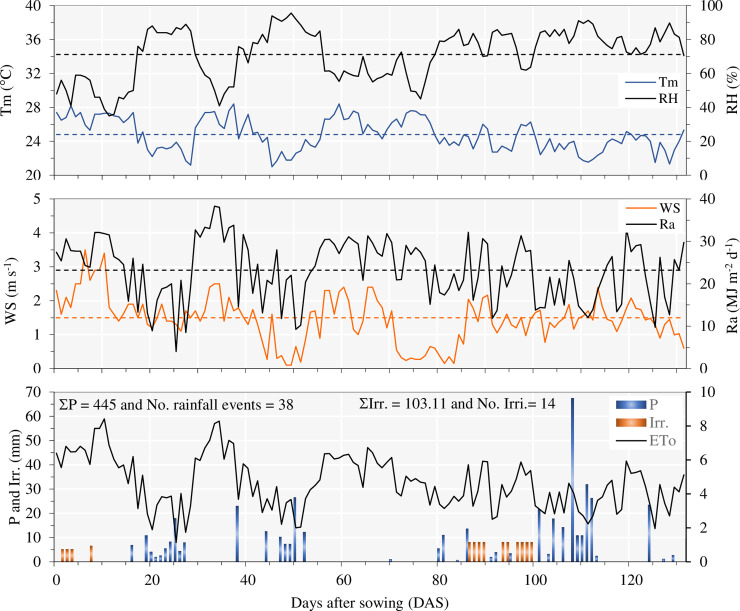
Meteorological data during the soybean growing season 2016/17. Tm: mean air temperature; RH: relative humidity; WS: wind speed at 2 m height; Ra: extraterrestrial radiation; P: rainfall; ETo: grass reference evapotranspiration; Irr.: irrigation depth. The dashed line represents the average of the data in the season.

The seasonal meteorological data during the 2016/17 soybean growing season are presented in Fig 2. The average air temperature over the crop season was 24.8°C, with maximum and minimum temperatures of 28.4 and 21.0°C, respectively. The average temperature remained slightly below that of the climatological normal for the region, which in this period of the year is 25.98°C [33]. The highest temperatures and the highest irrigation demand (high evapotranspiration) corresponded to the summer months (December to March), which also experienced considerable rainfall events.

In relation to rainfall, during the 2016/17 soybean season (Oct. 24, 2016, to March 04, 2017), there were 38 rain events, totalizing 445 mm (Fig 2); consequently, irrigation is predicated as a supplementary practice in this period, which is different for the winter plantings when the full cycle is irrigated. The irrigation was performed only 14 times, with a total irrigation depth applied of 103.1 mm. (Fig 2.). The ETo had an average value of 4.65 mm d^-1^ with some peaks in early and late November, which reached values higher than 8 mm. Much attention must be given when this high ETo peak occurs to avoid deficient irrigation depth that can, as a consequence, cause water stress in the plants. For example, stress during node emergence delays node appearance and hastens the formation of reproductive organs on these nodes, while if the plants are subjected to stress during flower and pod formation, they have a shorter period in which organs appear [52].

As the EEFlux is an energy balance-based model [16,18], the climatic conditions have a lot influence on their analysis, specially the solar radiation variable, main component of energy balance. Another point is that EEFlux requires identification of a hot and cold pixels in the image, thus, in the wet season or in irrigated areas the hot pixel can be hard to be identify, leading to inconsistent results. However, according to o Floolad et al. [16], in the EEFlux, these pixels are determined automatically with a great effectiveness in agricultural areas, generating ETrF and ETa values comparable to the values from trained expert to choose these pixels.

### Within-field variability of NDVI, ETa and Kc

**Fig 3:**
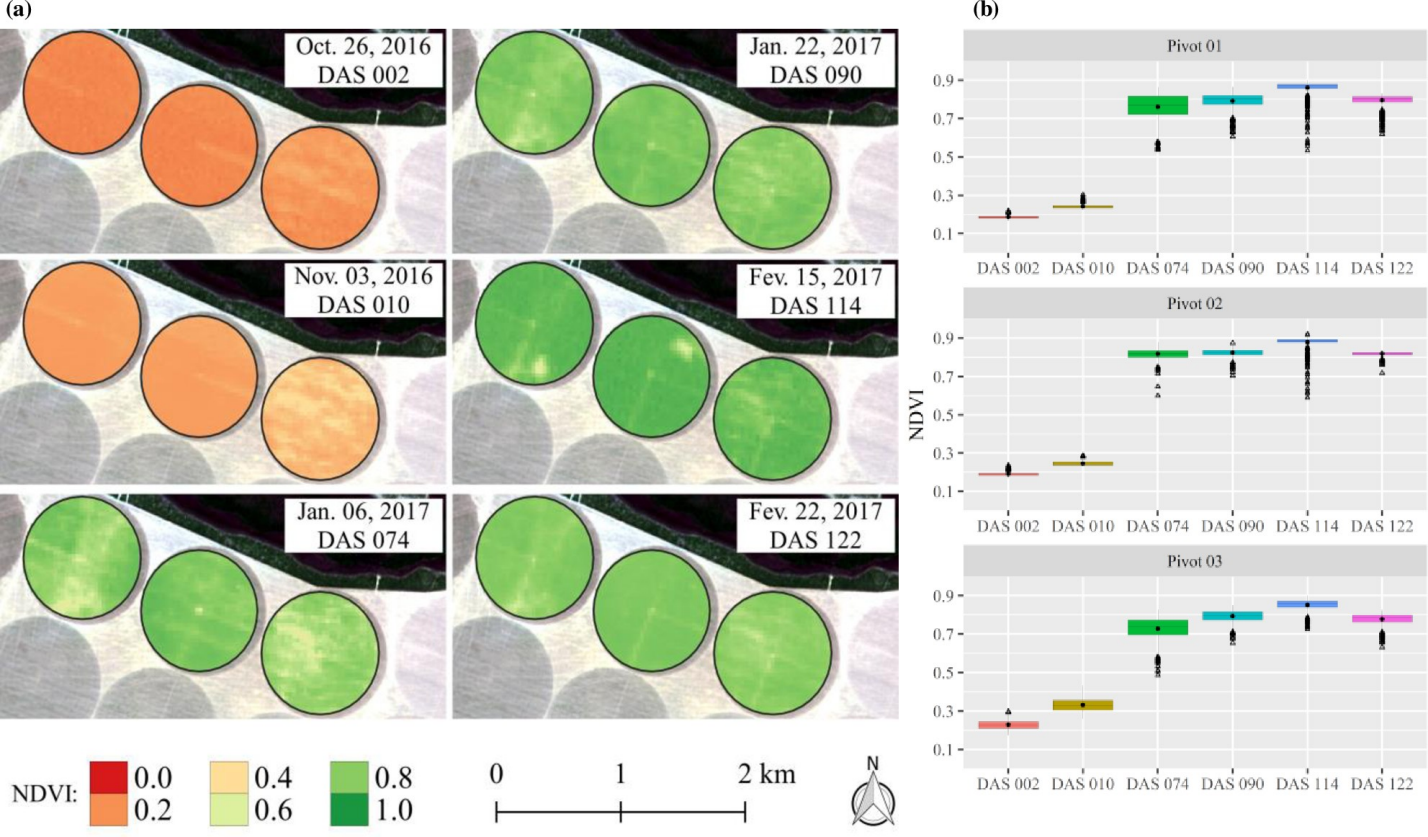
Spatiotemporal distribution (a) and boxplot (b) of the normalized difference vegetation index (NDVI) as a function of days after sowing (DAS) in the 2016/17 soybean growing season.

Understanding patterns of vegetation based on spectral vegetation indices (VI) is essential for crop management and to help farmers make decisions [40]. Fig 3A shows the spatiotemporal distribution of NDVI over the soybean growing season. As expected for normal soybean cultivation, low NDVI values were found in the initial stages (0 and 010 DAS) due to the larger amount of uncovered soil in this period. The emergence of soybeans usually occurs 5 to 10 days after sowing, depending on moisture and temperature conditions [53,54]. According to González-Gómez et al. [55], during the crop implantation phase, the vegetation index responses are mainly attributable to the bare soil or the remaining previous crop in direct sowing fields.

An expressive increase in NDVI during the development phase was verified, which can be clearly attested to by the difference between images at 010 and 074 DAS (Fig 3A and 3B). During the mid-season (074 and 090 DAS) and initial part of the late season (144 DAS), a low variability in the values was observed, and in the final portion of the late season (122 DAS) (Fig 3A), a reduction in the NDVI values until physiological maturity due to plant senescence was noticed [53]. It is important to note that this soybean cultivar (Monsoy—M8349 IPRO) presents a cycle of approximately 140 days in the study region; thus, at 122 DAS, the soybean still presents a high NDVI value (approximately 0.8) (Fig 3B). However, from this time, NDVI tends to decrease very fast until the R8 stage, where full maturity is reached– 95% of the pods have reached their mature pod color [53,54].

With 074 DAS, zones with low NDVI values were identified in central pivots 1 and 3, while in central pivot 2, we observed a great uniformity in NDVI values, that is, low data variability. The explanations for this are soil patches and failures in fertilization applications, since pests and diseases in the fields were not observed. On the other hand, in the last three images, a significant variability in NDVI values was not found. NDVI values during the season ranged from 0.2 to 0.9 (Fig 3A), which is a very common variation in soybean fields [56–59], with average rates always higher than 0.7 in the development, mid-season and late-season phenological phases. The small amplitudes of the boxplots reveal that vegetation conditions were slightly variable between the three fields over the growing season. This is a consequence of the high technological level adopted on the farm and of the use of only one cultivar.

**Fig 4:**
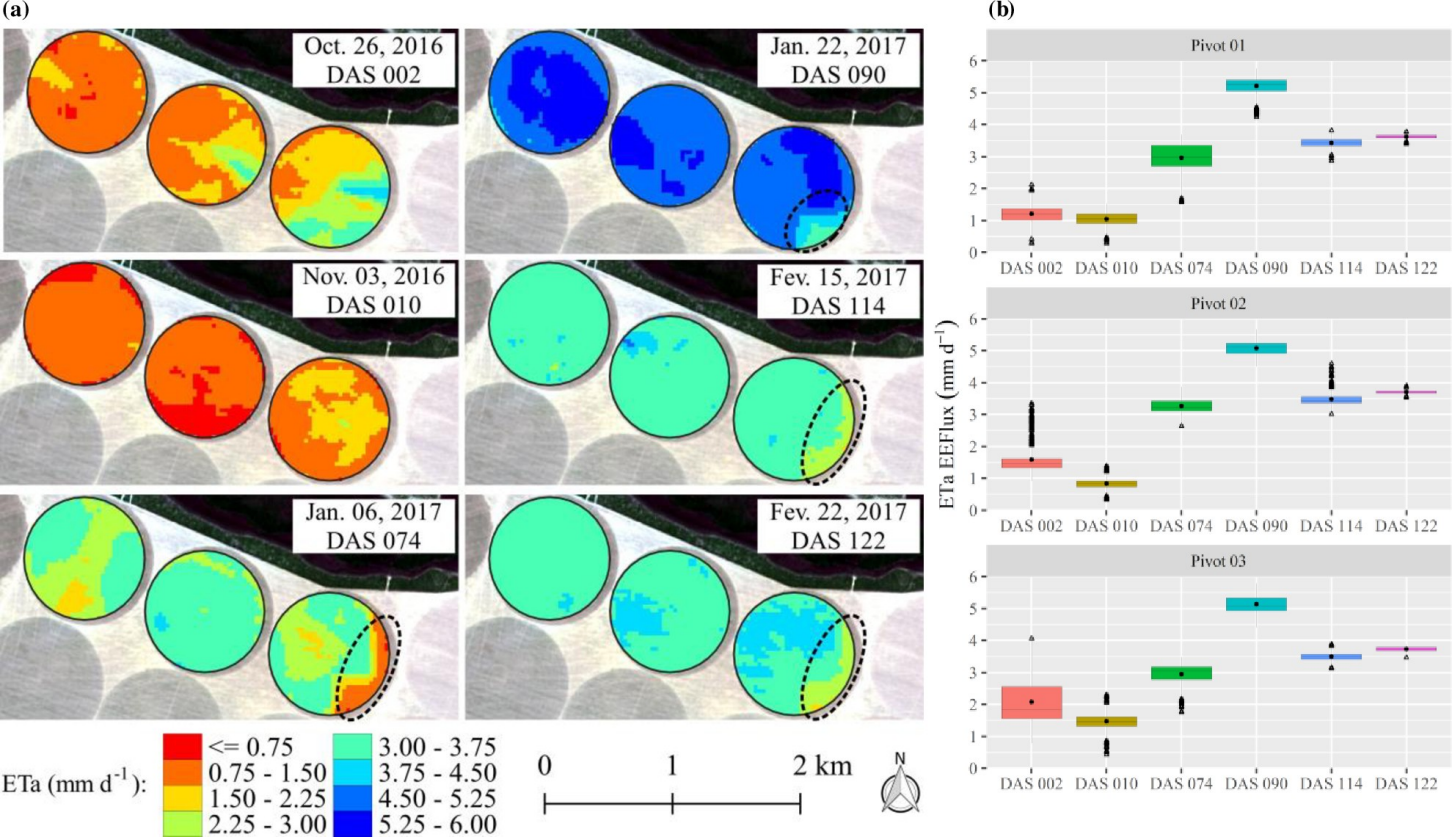
Spatiotemporal distribution (a) and boxplot (b) of the soybean actual evapotranspiration (ETa) values as a function of the days after sowing (DAS) in the 2016/17 soybean growing season. The area inside of the dashed line refers to the part considered affected.

The spatial and temporal variability of the soybean ETa from EEFlux over fields are presented in Fig 4A. In the first image (002 DAS), the major variability among the six images considered was verified, highlighting pivot 3 with two well-distinct zones, which can also be attested to with the boxplot amplitude. The two zones are a consequence of irrigation in a slice of the central pivot (east zone). Similar results were verified for central pivot number 2. The higher ETa value is verified at 090 DAS and is highly influenced by the high leaf area index, which normally has a maximum value at approximately 80 DAS [60,61]. The ETa remained relatively high in the subsequent images (114 and 122 DAS), and with respect to ETa uniformity, the greatest uniformities were observed in the three last images as a consequence of the good crop establishment in the area.

The boxplot of ETa values for the central pivots cultivated in the 2016/17 growing season are shown in Fig 4B. The average ETa values ranged from 1 to 5 mm d^-1^ considering the three fields. In addition to the NDVI, the ETa presented a seasonal behavior very characteristic of annual crops, that is, low values at the start of the crop season, maximum values in the mid-season and decreased values in the late season. The detection of within-field variability by satellite or remotely piloted aircraft system (RPAS) images can be very useful for precision irrigation because this approach can provide specific information about irrigation, such as water application uniformity, areas with irrigation deficits or surface runoff areas near the outer boundary of the central pivot systems. For Campos et al. [62], the variable application of agronomic inputs (e.g., water using variable-rate irrigation by central pivot) is an obstacle to precision agriculture, and variability maps can be essential for overcoming these obstacles because they provide accurate information about the real demand of each field zone.

**Fig 5:**
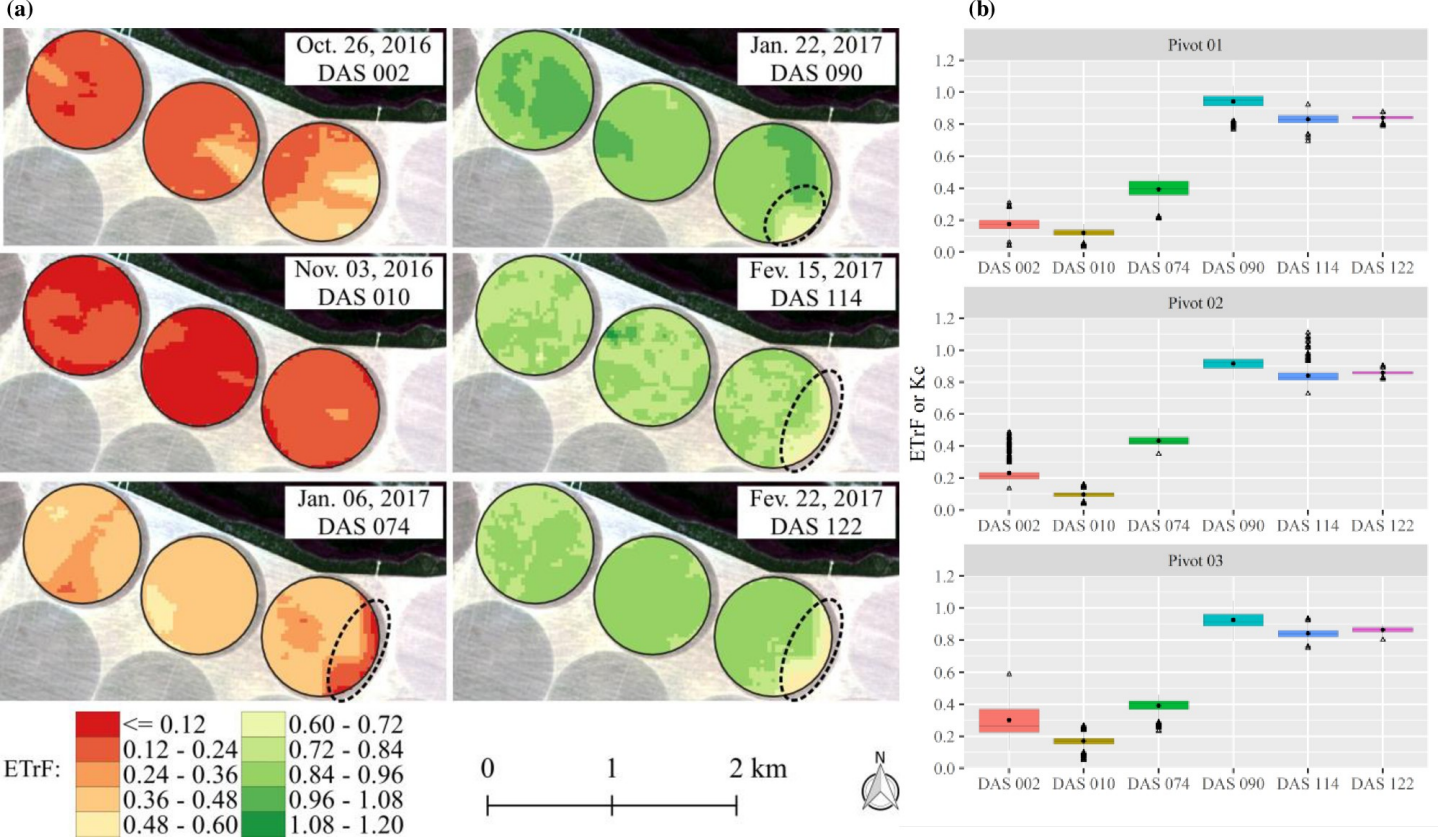
Spatiotemporal distribution (a) and boxplot (b) of the fraction of the reference evapotranspiration (ETrF) or alfalfa-based crop coefficient (K_c_) values as a function of the days after sowing (DAS) in the 2016/17 soybean growing season. The area inside of the dashed line refers to the part considered affected.

It is important to note that the ETa and ETrF EEFlux products presented inconsistency in some parts of pivot 3 (area within the dashed line, Figs 4A and 5A); specifically, patches of known geometry appeared—a kind of oversize pixel—very different from the Landsat 30 m-pixel (S1–S3 Figs). In addition, low values were verified in these areas when compared to the non-affected zones. A specific answer to this issue was not found in the literature, but it strongly looks like a simple processing error of the gridded weather data required for their calibration and calculation (e.g., of the ETa and ETrF), which show very low spatial resolution. In the present study area and in all other areas outside of the continuous United States, EEFlux uses Climate Forecast System Version 2 (CFSv2) and the Climate Forecast System Reanalysis (CFSR) gridded weather data for all calculations [16]. Another point that supports this hypothesis is that NDVI images, which do not require these climatic data, do not present the same issue. These affected zones were not considered in the statistical analyses.

The spatiotemporal evolution of the K_c_ for the three central pivots with soybeans followed a well-defined pattern featuring four different phases, such as crop implantation, fast growth, peak, and decrease, as also defined in FAO 56 (Fig 5A and 5B). It is verified that K_c_ ranged from approximately 0.10 to 1.0 considering mean values and all center pivots (Fig 5B) and, as expected, the highest values occurred in the mid-season (in this case, 090 DAS), when the soybeans have a maximum leaf area index and a higher vigor, as previously referenced. This K_c_ amplitude follows the findings presented by Kamble et al. [63]. An important detail (also occurring in ETa) is that the K_c_ value at 010 DAS is lower than 002. The most important reason for these results was the irrigation performed in the two first-day cycles (commonly called “irrigation for germination”), which made the soil more moist and consequently increased the K_c_ EEFlux, while in the two days previous to 010 DAS, the water (rain or irrigation) was not applied. Overall, the K_c_ presented a good spatial distribution pattern, which was close to the pattern found for ETa. This good spatial pattern as well as the convenient K_c_ values enables the combination with a traditional irrigation management system, such as the MFAO and FAO 56 approaches.

### Comparison of the ETa and Kc EEFlux with the MFAO

**Fig 6:**
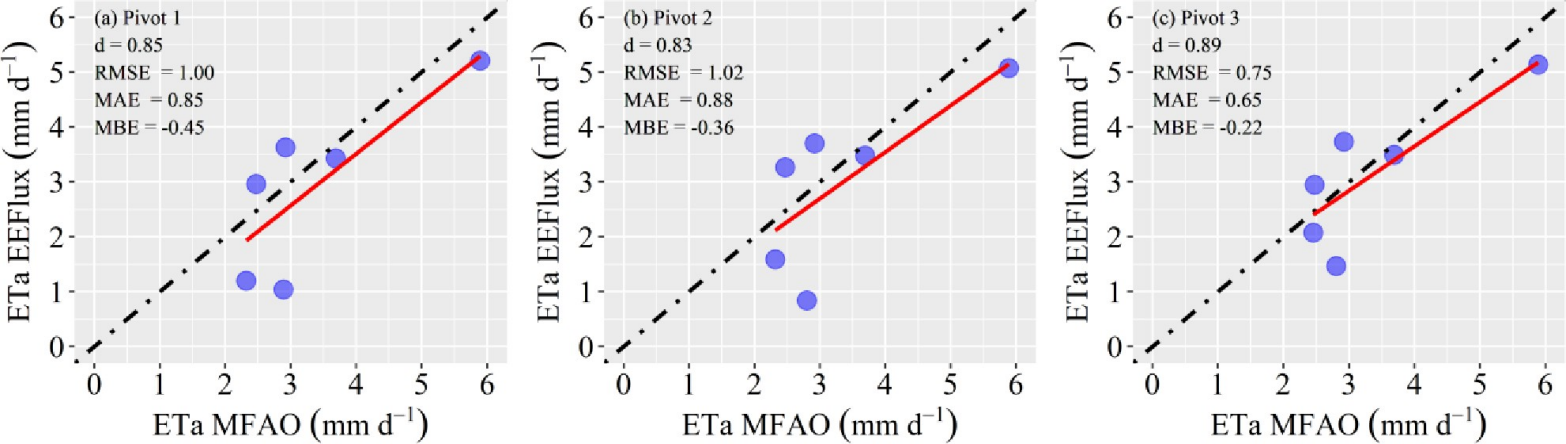
Comparison scatterplot between the average daily ETa from the MFAO approach and the ETa estimated by the EEFlux algorithm across the three central pivots in the 2016/17 soybean growing season. The dot-dashed line represents the 1:1 line, and the solid red line represents the linear regression.

The scatterplots in Fig 6 show comparisons between the average daily ETa from the MFAO approach and the ETa estimated by the EEFlux algorithm across the three central pivots in the 2016/17 soybean growing season. The corresponding values for the d-*index* were 0.85, 0.83 and 0.89 for pivots 1, 2 and 3, respectively, indicating a very good correlation between the ETa MFAO and ETa EEFlux. On the other hand, taking into account the RMSE, the difference between ETa EEFlux and MFAO was large in pivots 1 and 2, with values close to 1.0 and slightly smaller in pivot 3 (0.75 mm d^-1^). MAE was also higher for pivots 1 and 2 than that in field 3. It can also be deduced from MBE (Fig 6) that EEFlux tends to underestimate the ETa, especially when MFAO presents low(er) ETa values, as occurs in the initial phenological phases.

Overall, the comparison (using MFAO as reference) results were very satisfactory once MFAO data were updated and EEFlux incorporated the variability in the fields. A second point can be related to the use of an average Kc value in the MFAO during the initial and mid-season phases [6], while EEFlux, as well as other evapotranspiration models, takes into account the current conditions at the satellite overpass time. Another point is that the MFAO evapotranspiration was based on grass reference evapotranspiration, while EEFlux was calculated using the alfalfa reference, which can also contribute to the difference that was verified. Last, there are also limitations related to the number of samples available within the crop cycle, where the small number of samples tends to decrease the precision and accuracy of ET estimation models [17].

**Fig 7:**
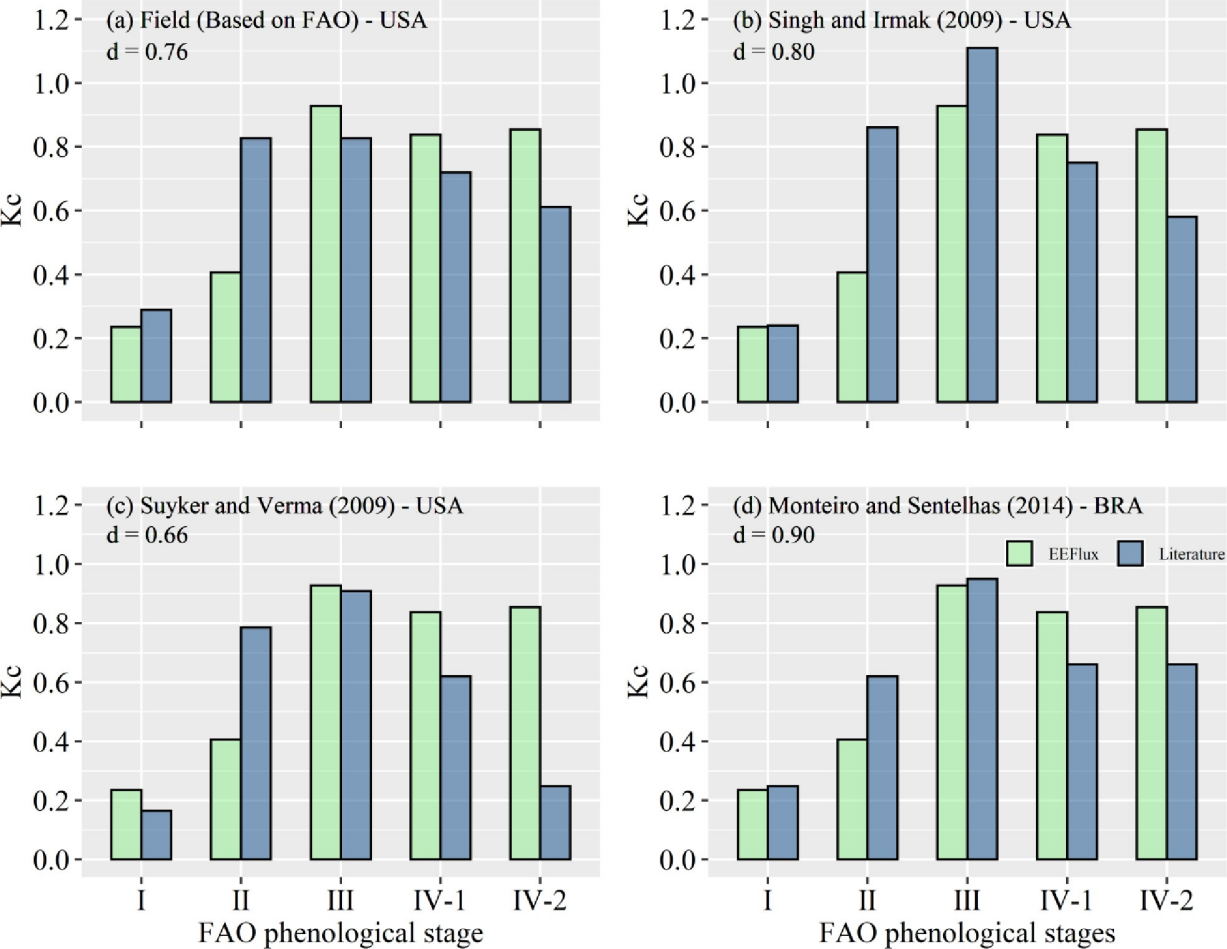
Soybean crop coefficient (K_c_) derived from EEFlux compared with the K_c_ adopted in the soybean fields and with those derived from studies performed in the United States and Brazil. The numbers 1 and 2 after phase IV indicate that the two images were acquired within this phase (see Table 5).

The comparison among the average values of the soybean K_c_ derived from EEFlux with the K_c_ adopted in the soybean fields and those derived from studies performed in the United States and Brazil are presented in Fig 7. The K_ratio_ value obtained to convert Kc-grass into Kc-alfalfa was 1.21, which is very close to the value recommended for semiarid regions, which is 1.20 [6]. First, it is important to highlight that K_c_ EEFlux agrees very well with the crop-development phases; that is, there are lower values during the initial period (phase I), a substantial increase in phase II in comparison to that of the previous phase, maximum values reached in mid-season (phase III) and a decrease in values in the last phase (IV). The average K_c_ EEFlux values obtained from soybean were 0.24, 0.41, 0.93, 0.84 and 0.85 for I, II, III, IV-1 and IV-2, respectively.

The comparison between K_c_ EEFlux and K_c_ used in the soybean fields, which is based on those values presented in the FAO 56 bulletin [6] can be observed in Fig 7A. There were verified close values for phases I, III, IV-1 and IV-2 and a higher difference in phase II, where K_c_ FAO values were two times higher than those of K_c_ EEFlux. Similar results were verified for the comparison with Singh & Irmak [47] data (Fig 7B) and Suyker & Verma [48] (Fig 7C). In addition to presenting a large difference in phase II, the results of Suyker & Verma [48] also showed a large difference in phase IV-2. The comparison of the K_c_ EEFlux to the K_c_ determined by Monteiro & Sentelhas [49] showed the best agreements (d = 0.90) (Fig 7D) and also showed a lower K_c_ difference in phase II, which did not occur in the other comparisons.

Costa et al. [17], in a very similar study, compared K_c_ EEFlux to K_c_ derived from FAO56 for maize crops, and according to them, the difference between K_c_ values is related to the temporal variability. Once the K_c_ FAO is obtained from multiday data, the K_c_ EEFlux is obtained from the passing time data of the satellite, which makes these methodologies considerably different. Another two points to be considered are the edaphoclimatic conditions and the methodologies used to obtain the K_c_, which possibly are different from those in the current study (satellite data with 30 m spatial resolution). Overall, K_c_ EEFlux represented soybean development well, which makes these products an important possibility for precision irrigation practices.

### NDVI-K_c_ relationship

**Fig 8:**
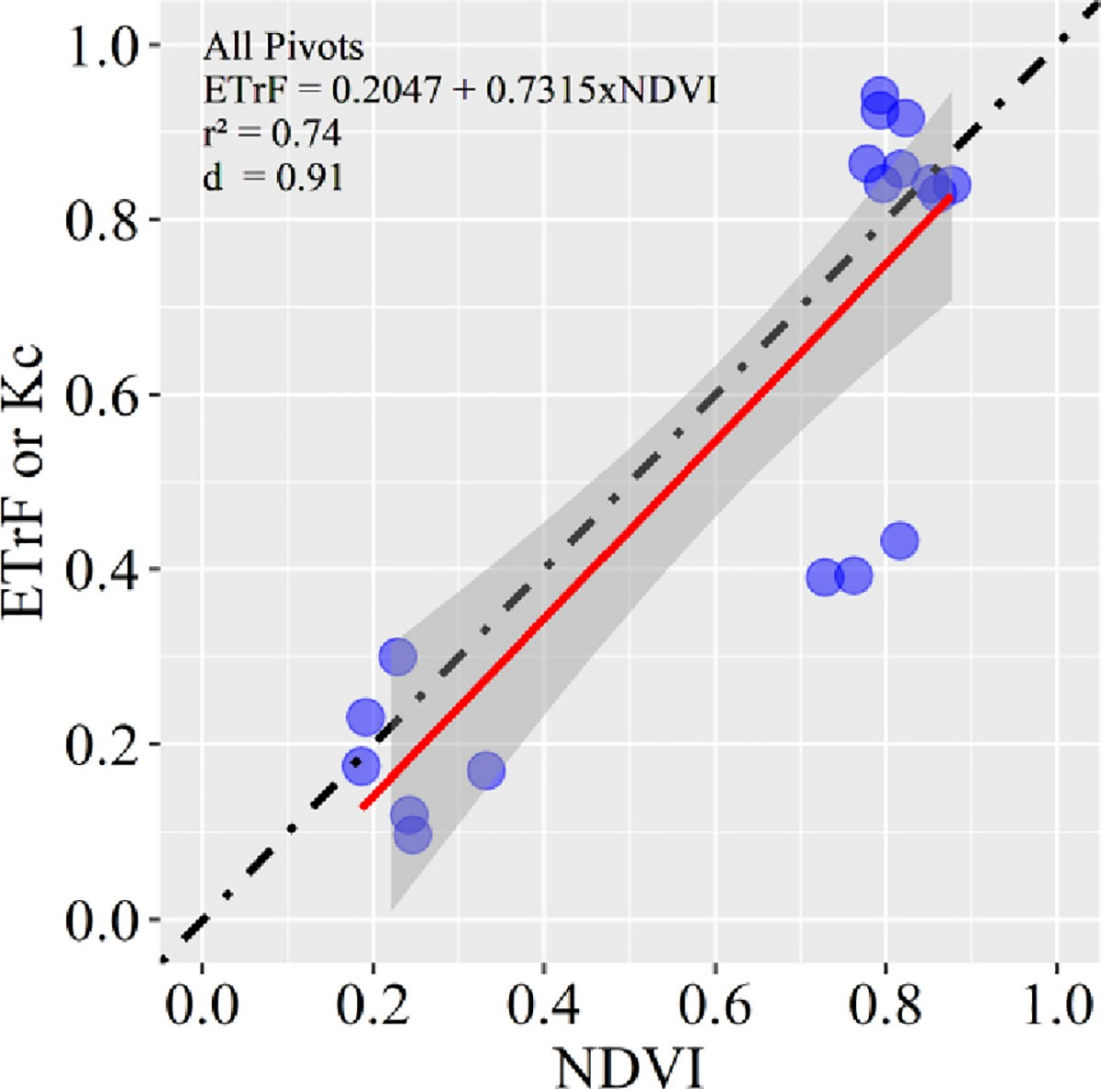
Linear relationship between NDVI and ETrF or K_c_ using data of all three central pivots with soybeans in the 2016/17 growing season. The smoothed area represents the 95% confidence interval. The dot-dashed line represents the 1:1 line, and the solid red line represents the linear regression.

The relationship between NDVI and K_c_ for irrigated soybean crops in the 2016/17 growing season is shown in Fig 8. There was a good correlation between NDVI and the K_c_, with a coefficient of determination (r^2^) equal to 0.74, which is very informative regarding the amount of variance that NDVI can explain in the K_c_ dataset. The d-*index* is 0.91, which means there is very good agreement between the datasets. An interest in estimating the K_c_ with the vegetation index, mainly to generate information for irrigation scheduling, appears with the availability of high spatial and temporal resolution multispectral satellite systems and the emergence of ultrahigh spatial resolution aerial platforms such as RPAS [64]. This fact would help precision irrigation practices, especially at a regional scale.

The isolated group with three NDVI values of approximately 0.8 and K_c_ of 0.4 is related to the image corresponding to 074 DAS (Figs 3B and 5B), where NDVI (depending only on vegetation) is high (≈ 0.8), while ETrF, which depends on more factors (e.g., soil moisture and climatic conditions), remained at approximately 0.4. One of the first works developed with this objective was performed by Bausch and Neale [65], and since then, numerous studies have found good results in estimating vegetation index-based K_c_ using linear regression models in different agricultural areas around the world [63,64,66,67].

### Soybean WP

Table 6 shows the soybean water productivity (WP) on two bases, calculated from the ETa estimated by MFAO methodology, and harvest machine-measured yield data. The sums of soybean evapotranspiration (ETa) estimated by MFAO methodology (sum ETa MFAO) were 361.5, 360.9 and 344.3 mm for central pivots 1, 2 and 3, respectively, while the average soybean yield was 4,042 kg ha^-1^. These yield values were very close to the mean value of the region, which was 3,960 kg ha^-1^ [31], and the yield is considerably higher when compared to Brazilian mean values, which were 3,206 kg ha^-1^ in the 2018/19 season [68]. The WP values were very close for the three central pivots, with a mean value of 1.14 kg m^-3^ or 11.37 kg ha^-1^ mm^-1^. In the southern Brazil region, the attainable water productivity found for soybeans was 9.1 kg ha^-1^ mm^-1^ [69]. In the USA western Corn Belt region, an estimated soybean attainable water productivity was 9.9 kg ha^-1^ mm^-1^ [70]. These references support our current results.

**Table 6:** Water productivity (WP) for the central pivots in the 2016/17 soybean growing season.

Central Pivot	Sum ETa MFAO (mm)	Sum ETa MFAO (m^3^ ha^-1^)	Yield (kg ha^-1^)	WP (kg ha^-1^ mm^-1^)	WP (kg m^-3^)
**1**	361.5	3,615.4	4,042	11.18	1.12
**2**	360.9	3,609.4	4,042	11.20	1.12
**3**	344.3	3,443.0	4,042	11.74	1.17
**Mean**	355.6	3,555.9	4,042	11.37	1.14

On the other hand, we found values higher than the values found by Alfonso et al. [71] in Balcarce, Argentina. These authors evaluated the water productivity in soybeans, following a cover crop in a humid environment, and the highest value found was 8.3 kg ha^-1^ mm^-1^, while the mean of all treatments was 7.23. It is important to note that the WP can suffer considerable variations according to irrigation management. For example, Gajić et al. [72] worked with four irrigation treatments (full irrigation, 65% of full irrigation, 40% of full irrigation and nonirrigated) during three experimental seasons and verified that under 65% of full irrigation, the soybean WP was 1.74 kg m^-3^, while under full irrigation, it was only 0.59 kg m^-3^. These results highlight the importance of studies related to controlled deficit irrigation to increase water use efficiency in agriculture.

## Conclusions

Precise information about the spatiotemporal variability of actual crop evapotranspiration (ETa) and crop coefficient (Kc) is crucial for water efficient management in the agriculture, which is the largest user of freshwater in the world. Remote sensing models has a lot potential to be used for this purpose. Thus, we used EEFlux, a METRIC version that operates on the Google Earth Engine (GEE) system, in commercial soybean cultivation in the municipality of São Desidério, State of Bahia, Brazil, to map within‑field variability of soybean evapotranspiration and crop coefficients.

Due to the lack of measured ET data, EEFlux modeled data were not validated but only compared to ET estimates by the MFAO method, which is a verified and utilized method in Brazil. Nevertheless, our findings confirm that the EEFlux platform, an innovative and free tool for access spatiotemporal variability of ETa and Kc at global scale is very efficient to estimate the ETa and Kc on different growth stages of soybean crop. The comparison between daily ETa estimated by the MFAO method and EEFlux showed good agreement for the three central pivots, exhibiting a d-*index* of 0.85, 0.83 and 0.89 for pivots 1, 2 and 3, respectively, but there is a subtle underestimation of ETa in comparison to that of the MFAO. Regarding the K_c_ of EEFlux, with the exception of phenological phase II, good concordance was verified between the K_c_ values considered in the comparison. In addition, the average WP equal to 1.14 kg m^-3^ indicates that water is being used with high efficiency in the soybean fields in the study.

ETa and K_c_ EEFlux are free access and ready-to-use, which makes them excellent data sources for the scientific community and other professionals involved in remote sensing in agricultural fields. However, in this study, we verified an anomaly related to the pixel oversize and low values of ETa and K_c_ in comparison to those in the non-affected zones. Further studies are necessary to correctly identify this issue. Last, ETa is a very dynamic phenomenon that depends especially on crop development, water supply and climatic conditions. Thus, the acquisition of a minimal number of images evenly distributed over the growing season for the assessment of spatial variability is very important. In this study, the six images used were able to characterize the variability in irrigated soybean cultivation once they were well distributed within the phenological phases.

## Supporting information

S1 Fig Natural color (RGB 432), false color (RGB 543), actual crop evapotranspiration (ETa), and fraction of the reference evapotranspiration (ETrF) referring to the image of the Landsat 8 satellite of 2017/01/06 when the soybean was with 074 days after sowing. The natural color and false color images show that there are no problems in the soybean fields. Thus, this strengthens our hypothesis that the error verified in the ETa and ETrF of the EEFlux is a consequence of the processing of the gridded weather data required to their calibration and calculation.
(DOCX)

S2 Fig Actual crop evapotranspiration (ETa) referring to the image of the Landsat 8 satellite of 2017/01/06 when the soybean was with 074 days after sowing. A large area around the central pivots studied was selected to demonstrate the EEFlux error. The area inside of the dashed line refers to the part considered affected.
(DOCX)

S3 Fig Fraction of the reference evapotranspiration (ETrF) referring to the image of the Landsat 8 satellite of 2017/01/06 when the soybean was with 074 days after sowing. A large area around the central pivots studied was selected to demonstrate the EEFlux error. The area inside of the dashed line refers to the part considered affected.
(DOCX)
