# Supplementary material for: Mapping within‑field variability of soybean evapotranspiration and crop coefficient using the Earth Engine Evaporation Flux (EEFlux) application
Source: PLoS One. 2020 Jul 9;15(7):e0235620. doi: 10.1371/journal.pone.0235620 (PMC7347170; doi:10.1371/journal.pone.0235620)
Supplement: S1 Fig — The natural color and false color images show that there are no problems in the soybean fields. Thus, this strengthens our hypothesis that the error verified in the ETa and ETrF of the EEFlux is a consequence of the processing of the gridded weather data required to their calibration and calculation. (DOCX) [file pone.0235620.s001.docx]

**
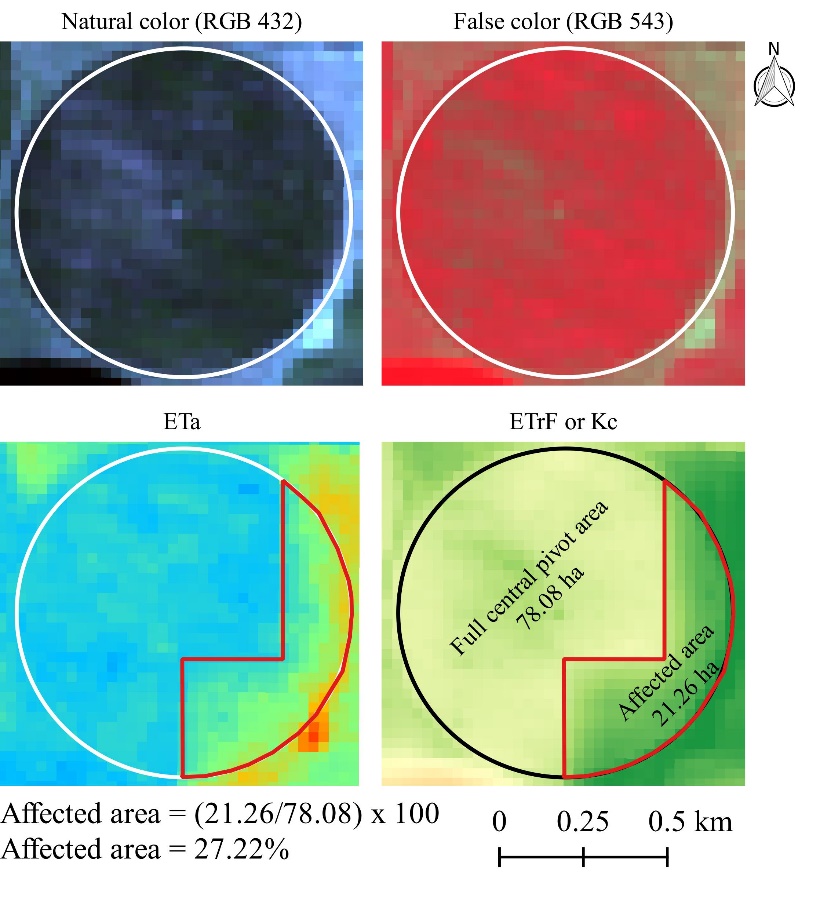
**

**S1 Fig. Natural color (RGB 432), false color (RGB 543), actual crop evapotranspiration (ETa), and fraction of the reference evapotranspiration (ETrF) referring to the image of the Landsat 8 satellite of 2017/01/06 when the soybean was with 074 days after sowing.** The natural color and false color images show that there are no problems in the soybean fields. Thus, this strengthens our hypothesis that the error verified in the ETa and ETrF of the EEFlux is a consequence of the processing of the gridded weather data required to their calibration and calculation.
