# Supplementary material for: Mapping within‑field variability of soybean evapotranspiration and crop coefficient using the Earth Engine Evaporation Flux (EEFlux) application
Source: PLoS One. 2020 Jul 9;15(7):e0235620. doi: 10.1371/journal.pone.0235620 (PMC7347170; doi:10.1371/journal.pone.0235620)
Supplement: S2 Fig — A large area around the central pivots studied was selected to demonstrate the EEFlux error. The area inside of the dashed line refers to the part considered affected. (DOCX) [file pone.0235620.s002.docx]

**
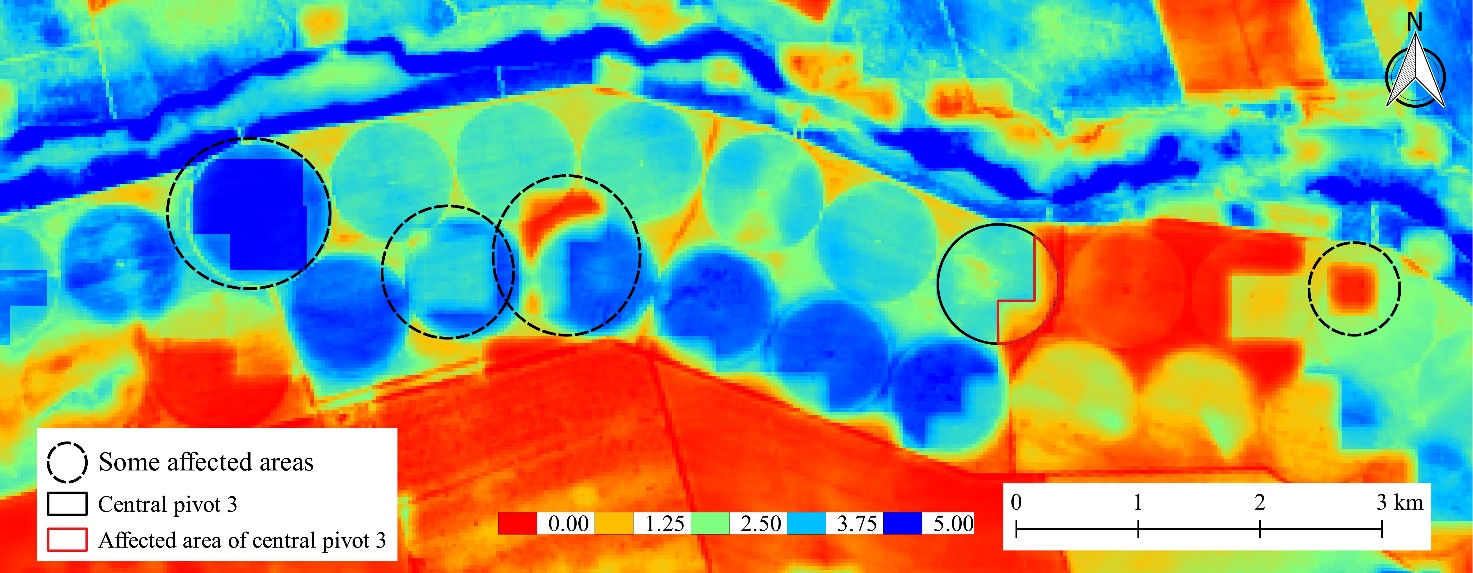
**

**S2 Fig. Actual crop evapotranspiration (ETa) referring to the image of the Landsat 8 satellite of 2017/01/06 when the soybean was with 074 days after sowing.** A large area around the central pivots studied was selected to demonstrate the EEFlux error. The area inside of the dashed line refers to the part considered affected.
